# Transcriptional control of CD4 and CD8 coreceptor expression during T cell development

**DOI:** 10.1007/s00018-013-1393-2

**Published:** 2013-06-21

**Authors:** Wilfried Ellmeier, Lena Haust, Roland Tschismarov

**Affiliations:** Division of Immunobiology, Institute of Immunology, Center for Pathophysiology, Infectiology and Immunology, Medical University of Vienna, Lazarettgasse, 19, 1090 Vienna, Austria

**Keywords:** T cell development, Coreceptor gene expression, Transcription factors, CD4/CD8 lineage decision

## Abstract

The differentiation and function of peripheral helper and cytotoxic T cell lineages is coupled with the expression of CD4 and CD8 coreceptor molecules, respectively. This indicates that the control of coreceptor gene expression is closely linked with the regulation of CD4/CD8 lineage decision of DP thymocytes. Research performed during the last two decades revealed comprehensive mechanistic insight into the developmental stage- and subset/lineage-specific regulation of *Cd4*, *Cd8a* and *Cd8b1* (*Cd8*) gene expression. These studies provided important insight into transcriptional control mechanisms during T cell development and into the regulation of *cis*-regulatory networks in general. Moreover, the identification of transcription factors involved in the regulation of CD4 and CD8 significantly advanced the knowledge of the transcription factor network regulating CD4/CD8 cell-fate choice of DP thymocytes. In this review, we provide an overview of the identification and characterization of CD4/CD8 *cis*-regulatory elements and present recent progress in our understanding of how these *cis*-regulatory elements control CD4/CD8 expression during T cell development and in peripheral T cells. In addition, we describe the transcription factors implicated in the regulation of coreceptor gene expression and discuss how these factors are integrated into the transcription factor network that regulates CD4/CD8 cell-fate choice of DP thymocytes.

## Introduction

The two major subsets of peripheral T cells express either the CD4 or CD8 coreceptor molecules. CD4-expressing cells constitute MHC class II-restricted helper T cells, while CD8-expressing T cells represent MHC class I-restricted cytotoxic T cells. CD4^+^ and CD8^+^ T cells develop in the thymus from so-called double-positive (DP) thymocytes that express both CD4 and CD8. Since the helper T cell phenotype is linked with CD4 expression and since cytotoxic T cells express CD8, it was hypothesized that the regulation of coreceptor gene expression is closely interconnected with CD4/CD8 cell-fate choice of DP thymocytes. Therefore, the characterization of the molecular details of how the expression of CD4 and CD8 is regulated was a promising approach to also obtain insight into the regulation of CD4/CD8 cell-fate choice. Indeed, the identification of the *cis*-regulatory networks driving *Cd4* and *Cd8* and the isolation of transcription factors regulating their expression provided not only fundamental insight into transcriptional control mechanisms during T cell development but also led to the identification of important members of the transcription factor network regulating cell-fate choice of DP thymocytes.

In this review, we first provide a brief overview about T cell development and CD4/CD8 cell-fate choice of DP thymocytes. We then describe the major *cis*-regulatory elements known to direct *Cd4*, *Cd8a*, and *Cd8b1* gene expression during T cell development and in peripheral T cells. Moreover, we present the transcription factors implicated in the regulation of coreceptor gene expression and discuss how these transcription factors are integrated into the transcription factor network that regulates CD4/CD8 cell-fate decision of DP thymocytes. Finally, we discuss recent advances in the three-dimensional control of nuclear positioning and interchromosomal interaction between the *Cd4* and *Cd8* loci. For a detailed in-depth description about the initial characterization of the various *Cd4* and *Cd8* *cis*-regulatory elements including the promoter regions, we refer to a previously published review [1].

## T cell development and models of CD4/CD8 lineage commitment

**Fig. 1:**
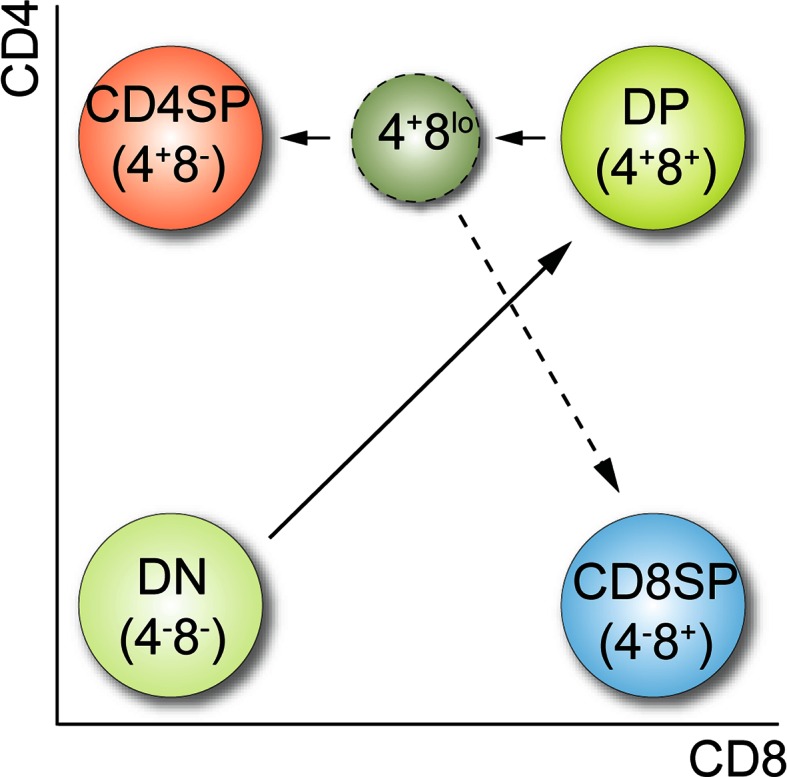
Simplified model of T cell development. Four major developmental stages of thymocytes can be distinguished based on CD4 and CD8 coreceptor expression: DN (CD4^−^CD8^−^), DP (CD4^+^CD8^+^), CD4SP (CD4^+^CD8^−^), and CD8SP (CD4^−^CD8^+^) thymocytes. DN cells develop into DP cells, which mature into CD4 and CD8SP thymocytes. At the onset of positive selection, DP thymocytes transcriptionally shut down CD8 expression, independently of whether they received MHC class I or class II signals. This results in the appearance of intermediate CD4^+^CD8^lo^ cells. According to the “kinetic signaling model”, CD4^+^CD8^lo^ cells expressing a MHC class II-restricted TCR have persisting TCR signaling and develop towards the CD4 helper T cell lineage (CD4SP). If CD4^+^CD8^lo^ cells express a MHC class I-restricted TCR, then TCR signaling is interrupted (due to the down-regulation of CD8). As a consequence, CD4^+^CD8^lo^ cells become susceptible to common cytokine receptor γ chain signaling (induced by IL-7 and Il-15), which leads to their specification into the CD8 cytotoxic T cell (CD8SP) lineage accompanied with to the cessation of CD4 expression and the re-expression of CD8 (“coreceptor reversal”) (*dashed arrow*). See text for more details. Adapted from [17]

The two major subsets of peripheral T cells express either the CD4 or the CD8 coreceptor molecules. CD4^+^ cells express a T cell receptor (TCR) specific for MHC class II and constitute the helper T cell population, while CD8^+^ T cells (which usually express CD8 molecules formed by heterodimers of the CD8α and CD8β chains encoded by the *Cd8a* and *Cd8b1* genes, respectively) express a TCR specific for MHC class I and represent the cytotoxic T cell pool. T cells develop in the thymus and the generation of CD4^+^ and CD8^+^ T cells is probably one of the best described developmental systems in mammals. During T cell development, the expression of CD4 and CD8 coreceptors is dynamically regulated and linked to the generation of helper and cytotoxic T cells. Based on the expression of CD4 and CD8, four major developmental stages of T cells can be distinguished (Fig. 1). So-called double-negative (DN) thymocytes that neither express CD4 nor CD8 define the earliest stages of developing thymocytes. At the DN stage, which can be further subdivided according the expression of CD44 and CD25, it is decided whether T cells develop towards the αβTCR or γδTCR T cell lineage. For cells committed to the αβT cell lineage, only those that have generated a functional TCRβ chain as a consequence of productive VDJ recombination of the *Tcrb* locus will proliferate and expand (the “β-selection” checkpoint), start to express CD8 and then CD4 and hence progress to CD4^+^CD8^+^ double-positive stage (DP) thymocytes. During the transition from the DN to the DP stage, cells also initiate the recombination of the *Tcra* chain locus, leading to the expression of a functional αβTCR complex on DP cells. At the DP stage, thymocytes undergo positive/negative selection events and CD4/CD8 cell-fate choice, resulting in the generation of CD4 and CD8 single-positive (SP) thymocytes that eventually exit the thymus to constitute the peripheral T cell pool. CD4 and CD8 contribute to this selection process by influencing the avidity of the TCR-self-peptide/MHC interaction, since CD4 and CD8 bind to the membrane-proximal invariant domains of the MHC class II and class I molecules, respectively [2, 3] and thus function as coreceptors. In addition, they influence the signaling function of the ligated TCR complex, since CD4 and CD8α bind to Lck (although with different affinities) via their cytoplasmic domains [4, 5]. The importance of the CD4 and CD8 coreceptor molecules is also demonstrated by the fact that CD4 or CD8-deficient mice have severely impaired helper or cytotoxic T cell development, respectively [6–8]. The positive/negative selection processes at the DP stage ensure the generation of a functional peripheral T cell pool with a diverse and broad specificity against foreign antigens presented by MHC molecules while potentially autoreactive T cells are deleted. For excellent and detailed reviews about several aspects of T cell development, we refer the reader to [9–12].

It was long-debated whether the TCR ligation in DP thymocytes by MHC at the onset of positive selection induces an instructive or a stochastic-selective signal that regulates CD4/CD8 cell-fate choice [13–16]. Experimental data supporting the “instructive model” of CD4/CD8 cell-fate decision suggested that TCR-MHC signals might quantitatively and/or qualitatively differ (in strength and duration) between MHC class II-signaled and MHC class I-signaled DP cells leading to termination of inappropriate coreceptor gene expression. However, data supporting the “stochastic-selective model” suggested that the initial lineage decision of DP thymocytes, characterized by the down-regulation of either CD4 or CD8, occurs independently of the MHC specificity (i.e., randomly) of the TCR expressed on DP thymocytes and is followed by a proof-reading mechanism that ensures that the T cell lineage matches the MHC-specificity of the TCR. A comprehensive overview of the various models and the experimental data supporting/conflicting them has been recently provided [17]. New insight into how CD4/CD8 cell-fate choice is regulated came from the observation that the kinetic of CD4 and CD8 expression in DP thymocytes is different in response to TCR–MHC interactions [18]. It has been shown that MHC-signaled DP thymocytes transcriptionally shut off *Cd8* but not *Cd4* gene expression, independent of whether DP cells received an MHC class I or class II signal [18]. The down-regulation of CD8 leads to the appearance of CD4^+^CD8^lo^ thymocytes, a key population of lineage uncommitted cells with the potential to develop towards either CD4 or CD8 lineage T cells [19, 20]. According to the “kinetic signaling model” proposed by Singer and colleagues [17, 18], CD4^+^CD8^lo^ cells expressing an MHC class II-restricted TCR continue to signal via their TCR, since the down-regulation of CD8 in MHC class II-signaled DP thymocytes does not interfere with the duration/strength of CD4/TCR-MHC class II engagement, leading to the development of CD4 lineage T cells. In contrast, for thymocytes expressing a TCR specific for MHC class I, the down-regulation of CD8 leads to an interruption of CD8/TCR-MHC class I signals. As a consequence of the cessation of TCR signals, CD4^+^CD8^lo^ cells become susceptible to common cytokine receptor γ chain (γc) signaling induced by IL-7 as well as IL-15, which is essential for the specification of the CD8 lineage fate [21, 22]. CD8 lineage specification of CD4^+^CD8^lo^ cells is accompanied by the termination of *Cd4* gene expression and the re-initiation of *Cd8* gene expression, a process termed “coreceptor reversal” (Fig. 1) [18]. Recently, the importance of the kinetic of *Cd4* and *Cd8* gene expression has been elegantly demonstrated by generating knock-in mice in which the *Cd4* gene was inserted into the *Cd8a* gene locus [23]. In these mice, MHC-signaled DP cells transiently down-regulated CD4 expression, and as a consequence, TCR-MHC class II signaling is interrupted. Indeed, as predicted from the kinetic signaling model, this led to the development of MHC class II-restricted CD4^+^ T cells with cytotoxic T cell properties. This demonstrates that thymocytes expressing CD4 with the kinetic of CD8 develop into cytotoxic T cells, thus providing convincing evidence that the kinetic of coreceptor gene expression is critical for CD4/CD8 lineage choice.

## *Cis*-regulatory elements controlling *Cd4* gene expression

**Fig. 2:**
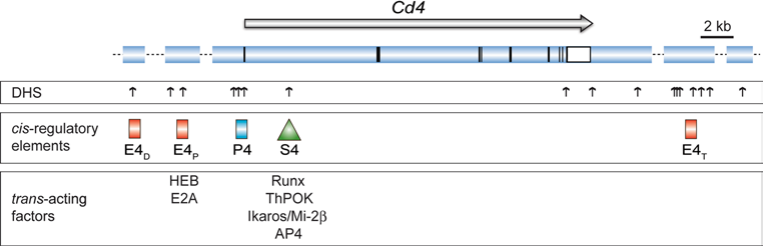
Map of the *Cd4* gene locus. The *upper panel* shows the *Cd4* gene locus and the *horizontal arrow* indicates the transcriptional orientation of the *Cd4* gene. *Vertical black* and *open bars/squares* indicate exons and the 3′ untranslated regions. *Vertical arrows* indicate DNase I hypersensitivity sites [24–26, 127]. The *red rectangles* indicate the location of *Cd4* enhancers E4_D_ (located approx. 24 kb upstream of the *Cd4* promoter), E4_P_ (13 kb upstream), and E4_T_ (20 kb downstream of the 3′ end), while the *triangle* indicates the location of the *Cd4* silencer (S4). Only those *trans*-acting factors are indicated where binding has been confirmed in primary thymocytes and T cells by chromatin immunoprecipitation assays (see text for more details). Drawing is adapted from reference [71]. The following references report binding to the *Cd4* gene locus: Runx complexes [73, 75], ThPOK [64, 128], Ikaros/Mi-2β [89], AP4 [92], HEB/E2A [93]

The murine *Cd4* locus maps within an 80-kb region on mouse chromosome 6 and shares a similar exon/intron structure with its human counterpart that is located on human chromosome 12. The mapping of DNase I hypersensitivity sites (DHS) around the murine *Cd4* locus and the subsequent testing of genomic fragments containing DHS for enhancer activity in a large number of transgenic reporter expression assays has led to the identification of several *cis*-regulatory elements that direct the expression of CD4 during T cell development [24–27]. These studies revealed the existence of several positive-acting *cis*-elements (i.e., *Cd4* enhancers) as well as the presence of a negative-acting *cis*-element, the so-called *Cd4* silencer (S4), in addition to the *Cd4* promoter (P4) (Fig. 2).

### *Cd4* enhancers display T cell-specific activities

The proximal *Cd4* enhancer E4_P_ maps approximately 13 kb upstream of the *Cd4* promoter and transgenic reporter assays and in vitro transfection experiments resulted in the identification of the 339 bp E4_P_ core fragment [25]. The generation of transgenic minigenes containing E4_P_, the *Cd4* promoter, and human CD2 as a reporter gene indicated that E4_P_ directs expression in all thymocyte subsets as well as in CD4^+^ and CD8^+^ T cells and thus that E4_P_ functions as a general T cell-specific enhancer. These data already indicated the existence of a negative-acting *cis*-element that counterbalances the activity of E4_P_ in CD8 lineage T cells (see below).

Two other *Cd4* enhancers are less well characterized. The so-called distal *Cd4* enhancer E4_D_ resides approximately 24 kb upstream of the *Cd4* promoter [28]. However, it is not clear whether this enhancer regulates *Cd4* expression or the expression of a neighboring gene (*Lag3*), which is located immediately upstream of E4_D_. In transgenic reporter expression assays, the combination of E4_D_ and E4_P_ resulted in the expression of the reporter gene not only in T cells but also in B cells and macrophages, suggesting that E4_D_ might not regulate *Cd4* [26]. Another element, the so-called thymic enhancer E4_T_, located 36 kb downstream of the *Cd4* gene, was shown to be necessary for efficient transgene expression in DP thymocytes [29].

### The *Cd4* silencer: a genomic fragment that restricts CD4 expression to helper lineage T cells

The transgenic reporter expression assays indicated that the helper-lineage-specific expression of CD4 is dependent on the presence of a region within intron 1 in the *Cd4* locus, which has been mapped to a 434-bp fragment designated as *Cd4* silencer. A similar *Cd4* silencer fragment has also been identified in the human *Cd4* gene locus [30]. While the expression of the transgenic reporter gene (hCD2 driven by E4_P_ and P4) in the presence of the *Cd4* silencer was restricted to T cells that express CD4 (i.e., DP and CD4SP thymocytes and CD4^+^ T cells), the removal of the *Cd4* silencer region from the transgenic reporter construct resulted in the expression of the reporter gene also in CD8 lineage T cells as well as in DN thymocytes [27]. These studies indicated that the *Cd4* silencer activity is dynamically regulated during T cell development. The *Cd4* silencer is active in DN thymocytes, inactivated during the transition to the DP stage, and reactivated specifically in thymocytes developing towards the cytotoxic lineage [27].

### Gene targeting approaches to study *Cd4 cis*-regulatory elements: evidence for epigenetic regulation of *Cd4* gene expression

The importance of the *Cd4* silencer in the regulation of *Cd4* gene expression was confirmed by gene targeting studies. The germline deletion of the *Cd4* silencer resulted in the derepressed expression of the *Cd4* gene both in DN thymocytes and in CD8 lineage T cells [31, 32], demonstrating the essential role for the *Cd4* silencer is the repression of CD4. By using a conditional *Cd4* silencer allele, Littman and colleagues [32] further showed that CD4 continued to be transcriptionally silent upon deletion of the *Cd4* silencer in mature CD8^+^ T cells, even if the cells underwent several rounds of proliferation. Together, these data indicate that the *Cd4* silencer is essential for the establishment of silencing during the commitment phase towards the CD8 lineage, but not for the maintenance once *Cd4* silencing has been established in peripheral CD8^+^ T cells. Mechanistically, the maintenance of *Cd4* silencing in the absence of the silencer appears to be independent of DNA methylation [32]. However, ChIP assays revealed that the repressive histone marks tri-methylation of histone 3 at lysine 9 (H3K9me3) or 27 (H3K27me3) are increased in the *Cd4* promoter region in CD8^+^ T cells [33], suggesting that these epigenetic histone marks might be involved in the stable repression of CD4. Further studies are required to determine how these marks at the *Cd4* promoter are established and whether they are indeed involved the regulation of *Cd4* silencing in CD8^+^ T cells. Interestingly, it has been shown that MHC class II-restricted CD4^−^CD8^+^ T cells that develop in ThPOK-null mice (as described in more detail below) up-regulate CD4 upon activation, indicating that the *Cd4* gene locus can be reactivated in redirected class II-restricted CD8^+^ cells in ThPOK-null mice [34]. This suggests differences in silencing mechanisms in redirected MHC class II-restricted CD8^+^ T cells that develop upon loss of ThPOK in comparison to wild-type MHC class I-restricted CD8^+^ T cells.

A crucial role for the *Cd4* enhancer E4_P_ for the initiation and maintenance of CD4 expression has been shown by generating a conditional “floxed” E4_P_ allele [35]. Deletion of E4_P_ was found to result in loss of CD4 expression in preselected DP thymocytes. However, a fraction of MHC class II-restricted E4_P_-deficient thymocytes could still be positively selected and, albeit at reduced levels, started to express CD4. This demonstrates that E4_P_ is essential for the early induction of *Cd4* gene expression in DP thymocytes and that E4_P_ is also required for high-level expression of CD4 in mature thymocytes and T helper cells. Moreover, these data also imply the presence of an additional *Cd4* *cis*-regulatory element that directs expression in mature T helper cells and this enhancer has been designated “maturation” enhancer [35]. However, mature CD4^lo^ E4_P_-deficient T cells lost CD4 expression upon activation and loss of CD4 expression was linked with cell proliferation after activation. This suggests that E4_P_ is necessary for the stabilization of CD4 expression upon activation. Interestingly, retroviral Cre-mediated deletion of the “floxed” E4_P_ allele revealed that CD4 expression remained at high levels when E4_P_ was deleted in mature T helper cells. The maintenance of CD4 expression correlated with active histone marks at the *Cd4* promoter region, suggesting epigenetic propagation of CD4 expression in the absence of E4_P_ [35].

In contrast to E4_P_, deletion of E4_T_ had no effect on CD4 expression in thymocytes or in peripheral mature T cells [35]. In addition, mice lacking both E4_P_ and E4_T_ had a similar phenotype as E4_P_-deficient mice, indicating that there is no compensatory role for E4_T_ in the absence of E4_P_ and that it is unlikely that E4_T_ represents the “maturation” enhancer mentioned above. However, E4_T_ is required for directing CD4 expression in a subset of lymphoid tissue inducer cells [35], indicating a subset-specific function for E4_T_.

Taken together, gene-targeting strategies revealed a crucial role for E4_P_ in the initiation of CD4 expression during thymocyte development and for high-level and stable expression of CD4 in T helper cells, while the *Cd4* silencer is essential for the establishment of *Cd4* silencing in CD8 lineage T cells. Remarkably, once mature T helper or cytotoxic T cells have been generated, *Cd4* expression or *Cd4* silencing can be maintained independently of E4_P_ or S4, respectively. This suggests epigenetic mechanisms controlling *Cd4* expression in mature T cells.

## *Cis*-regulatory elements controlling the *Cd8a* and *Cd8b1* gene complex

The CD8 coreceptor is usually expressed as a heterodimer formed by the CD8α and CD8β chains on conventional T cells. The CD8 chains are encoded by the *Cd8a* and *Cd8b1* genes, which are closely linked at a distance of about 36 kb on mouse chromosome 6 [36] and of about 15 kb on human chromosome 2 [37]. However, subsets of intraepithelial lymphocytes (IEL) in the gut [38, 39], CD8^+^ dendritic cells [40], and a subset of human NK cells [41] express CD8αα homodimers. This indicates both common regulatory mechanisms as well as independent transcriptional control of *Cd8a* and *Cd8b1* gene expression.

### A complex network of *cd8 *enhancers direct developmental stage- and lineage-specific expression of the *Cd8a* and *Cd8b1* genes

**Fig. 3:**
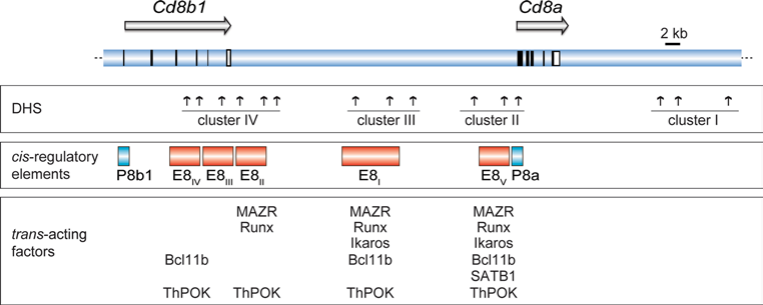
Map of the *Cd8ab* gene complex. The *upper panel* shows the *Cd8ab* gene complex and the* horizontal arrows* indicate the transcriptional orientation of the *Cd8b1* and *Cd8a* genes (after Gorman et al. [36]). *Vertical black* and *open bars/squares* indicate exons and the 3′ untranslated regions. *Vertical arrows* indicate DNase I hypersensitivity sites that are grouped into four clusters [42]. The *red rectangles* indicate the location of *Cd8* enhancers E8_I_–E8_V_. Only those *trans*-acting factors are indicated were binding has been confirmed in primary thymocytes and T cells by chromatin immunoprecipitation assays (see text for more details). Drawing is adapted from reference [71]. The following references report binding to the *Cd8ab* gene complex: MAZR [55], Runx complexes [75], Ikaros [108], Bcl11b [107], SATB1 [100], ThPOK [67]

Similar to the analysis of the *Cd4* locus, long-range DHS assays combined with transgenic reporter gene expression approaches were performed to identify *cis*-regulatory elements within the murine *Cd8ab1 (Cd8ab)* gene complex [42–44]. These studies resulted in the identification of at least five *cis*-regulatory elements within the *Cd8ab* gene complex that display a complex pattern of developmental stage-, subset-, and lineage-specific activities [42–47] (Fig. 3). The first *Cd8* enhancer identified was E8_I_, which directs expression of a reporter gene in mature CD8SP thymocytes, peripheral CD8^+^ T cells, and in CD8αα^+^ IEL [42, 43]. Subsequently, four additional enhancers were identified. E8_II_ is active in DP and CD8SP thymocytes and in CD8^+^ T cells, while E8_III_ is an enhancer whose activity is restricted to DP thymocytes [46]. E8_IV_ is active in DP thymocytes and CD8^+^ T cells, however E8_IV_ also directs low-level expression in CD4^+^ T cells [46]. The activity of E8_IV_ in the transgenic setting was attributed to the observation that approximately 25 % of CD4^+^ T cells express low levels of CD8β [45], although the biological function of CD8β expression in a fraction of CD4^+^ T cells is not clear. Finally, enhancer E8_V_ did not show any activity in transgenic reporter expression assays [45]. However if E8_V_ is used together with E8_I_ in a transgenic reporter construct, the combined activity of these two *Cd8* enhancers directed expression in DP thymocytes (in addition to mature CD8SP and CD8^+^ T cells as expected from the activity of E8_I_ alone), thus indicating combinatorial activity of *Cd8* *cis*-regulatory elements. Similar clusters of DHS have also been identified for the human *Cd8ab* gene complex and were shown to direct appropriate developmental expression in transgenic mice [48], and a cross-species comparison indicated several evolutionary conserved regions that overlap with DHS (Ellmeier, unpublished results), indicating a similar mode of *Cd8a* and *Cd8b1* gene regulation in man, mouse, rat, and dog.

The existence of *Cd8* enhancers that are either specifically active in DP cells (i.e., E8_III_; [46]) or in mature CD8 SP T cells (i.e., E8_I_; [42, 43]) provided also a potential molecular explanation for the transcriptional regulation of the *Cd8* genes during CD8 lineage differentiation, i.e., down-regulation of CD8 in signaled DP thymocytes (loss of E8_III_ activity) and “coreceptor reversal” and re-initiation of CD8 expression (due to the activity of E8_I_) during CD8 lineage differentiation [18]. Although the “switch of enhancer usage” model is very attractive to explain the dynamic expression of CD8, the generation of various *Cd8* enhancer-deficient mice revealed a more complex utilization of the various *cis*-regulatory elements at the endogenous *Cd8ab* gene complex during T cell development.

### Gene targeting approaches to characterize *Cd8* enhancer function

The identification of this complex regulatory mode mediated by the activity of several *Cd8* enhancer activities raised the question why so many *cis*-regulatory elements are required to direct expression of CD8. Several possibilities, which are not mutually exclusive, might be imagined. First, as briefly discussed above, *Cd8* enhancers might be used (in part) developmental stage-specific, thus facilitating the dynamic regulation of CD8 expression during T cell development. Second, some of the enhancers might be specific for directing *Cd8a* gene expression in conventional CD8^+^ T cells, while others might be specific for *Cd8b1*, a possibility that cannot be distinguished in transgenic reporter expression assays. Third, some of the enhancers displayed activity only in conventional T cells but not in IELs or in DCs and therefore might regulate the T cell subset-specific expression of CD8. Fourth, it is possible that the combined activity of all enhancers at the endogenous *Cd8* loci is required to direct high-level expression of CD8. Gene targeting approaches were used to delete *Cd8* enhancers to address the complexity of *Cd8* enhancers in more detail. The first enhancer deleted was E8_I_ [44, 46]. Despite its activity in mature CD8SP and CD8^+^ T cells in transgenic mice, in the absence of E8_I_ conventional DP thymocytes and peripheral CD8^+^ T cells express normal levels of CD8αβ heterodimers, with the exception of mature CD8SP thymocytes, which display a 20 % reduction of CD8 levels [46]. This suggests that loss of E8_I_ activity can be compensated by other *cis*-elements in conventional T cells. In contrast, there is a strong reduction of CD8α expression levels on γδTCR^+^ as well as on αβTCR^+^ IELs [44, 46]. These data indicate that E8_I_ is an enhancer essential for subset-specific expression of *Cd8a* in IELs. This was already implied by transgenic reporter assays, since E8_I_ is the only enhancer that directed expression in CD8αα^+^ IELs [43–46].

The generation of E8_II_-deficient mice revealed that E8_II_ is not essential for the regulation of the *Cd8ab* gene complex [49]. There was no detectable alteration in the expression of CD8 in conventional T cells or in IELs, suggesting that, like in the case of E8_I_, other *cis*-elements compensate for loss of E8_II_. Since E8_I_ and E8_II_ are both active in CD8^+^ T cells, E8_I_/E8_II_ double-deficient mice were generated to test whether E8_I_ and E8_II_ compensate for each other upon deletion [49]. Surprisingly, E8_I_/E8_II_ double-deficient thymocytes displayed variegated expression of CD8 in DP thymocytes, leading to the development of a fraction of “CD8-negative” DP thymocytes. A similar and even more enhanced population of “CD8-negative” DP thymocytes was also observed in E8_V_-deficient mice [50] and later also, although with a much weaker phenotype, in mice lacking E8_II_/E8_III_ [51]. The variegated expression of CD8 in E8_I_/E8_II_, E8_II_/E8_III_ and E8_V_-deficient thymocytes is conceptually reminiscent of position effect variegation observed in transgenic mice [52]. This suggested that the deletion of *Cd8* enhancers might lead to alterations in the chromatin structure of the *Cd8ab* gene complex and thus to an impaired activation of the *Cd8* loci during the DN-to-DP transition [53]. Interestingly, those DP thymocytes that were able to activate the *Cd8ab* gene complex expressed normal levels of CD8, indicating that other *cis*-regulatory elements maintain CD8 expression and/or that epigenetic mechanisms are involved in the regulation of CD8 expression in mature CD8^+^ T cells. The variegated expression in the absence of *Cd8* enhancers also implied that chromatin remodeling complexes might be recruited via these *Cd8* *cis*-regulatory elements and that the *Cd8ab* gene complex is epigenetically regulated [53]. Indeed, the BAF (Brahma associated factor) chromatin remodeling complex has been shown to contribute to the regulation of CD8 expression. Mice heterozygous for Brg1, the ATPase necessary for the activity of the BAF complex, display variegated expression of CD8 in DP thymocytes [54], suggesting a potential link between *Cd8* enhancers and the BAF complex. Moreover, subsequent studies in E8_I_/E8_II_-deficient mice have shown that CD8 variegation in DP thymocytes is accompanied with increased DNA methylation and reduced histone acetylation in cells that failed to upregulate CD8, indicating an “epigenetic block” [55]. The deletion of DNA methyltransferase (Dnmt) 1 in E8_I_/E8_II_-deficient thymocytes partially restored the activation of the *Cd8ab* gene complex [55], highlighting the role of E8_I_ and E8_II_ in recruiting factors that lead to epigenetic alterations at the *Cd8* loci.

## A transcription factor network links CD4/CD8 cell-fate choice and *Cd4*/*Cd8* coreceptor gene expression

**Fig. 4:**
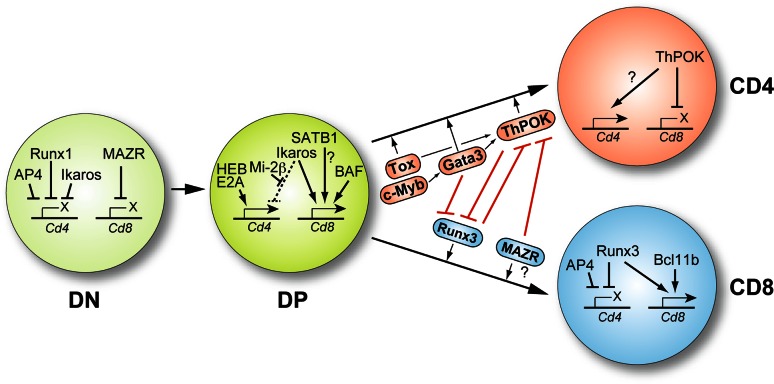
Transcriptional networks controlling coreceptor gene expression. Drawing showing the DN, DP, CD4SP, and CD8SP stages of T cell development. For each developmental step, the transcriptional regulators implicated in the positive and negative regulation of the coreceptors are indicated. For simplicity, the *Cd8a* and *Cd8b1* genes are shown as a single *Cd8* locus. In addition, the transcription factor network controlling CD4/CD8 cell-fate choice and the cross-regulation between CD4 lineage promoting factors (Tox, Gata3, ThPOK; shown in *red boxes*) and CD8 lineage factors (Runx3, MAZR; shown in *blue boxes*) is described. The repressive function of Ikaros on the *Cd4* gene is blocked by Mi-2β in DP cells (*dashed line*). The *question mark* indicates that it is currently not known whether MAZR affects CD8 lineage differentiation, in addition to the repression of *Thpok*, also by other mechanisms (see text for further details). Drawing is adapted from references [71, 129]

The identification of *cis*-regulatory elements directing the expression of the *Cd4* and *Cd8* genes provided the basis for experimental strategies to decipher the molecular nature of *trans*-acting factors regulating coreceptor gene expression. As described below, these experiments revealed that the Runx family of transcription factors is important for *Cd4* silencing and later studies also showed that Runx factors are essential for CD8 lineage differentiation. Together with studies that described the identification and characterization of the transcription factor ThPOK, which is a crucial regulator of CD4 lineage development, important insight into transcriptional control mechanisms regulating coreceptor gene expression during T cell development as well as into the transcriptional network coordinating CD4/CD8 cell-fate choice has been obtained (Fig. 4).

### ThPOK: a central regulator of CD4 lineage commitment

ThPOK (also known as cKrox; encoded by the *Zbtb7b* gene, referred to here as *Thpok*) is a member of the BTB/POZ domain-containing zinc-finger transcription factor family [56, 57]. ThPOK was identified by Kappes and colleagues [58] by uncovering the molecular cause of the phenotype of HD (helper-deficient) mice, which lack mature CD4^+^ T cells, and independently by Bosselut and colleagues [59] as a factor induced in DP thymocytes upon MHC class II-mediated positive selection that promotes CD4 lineage development. HD mice acquired a spontaneous point mutation in *Thpok*, leading to an amino acid exchange in the third Zn-finger domain of ThPOK [60]. MHC class II-restricted DP cells are still positively selected in HD mice, however they are redirected into the CD8 lineage, while MHC class I-restricted DP cells deficient for functional ThPOK develop normally into CD8 lineage cells [61]. On the other hand, transgenic overexpression of ThPOK on a HD-deficient background restored the development of CD4^+^ T cells and in addition resulted in the redirection of MHC class I-restricted DP thymocytes into the CD4 lineage [60]. Similar results were also obtained upon overexpression of ThPOK in wild-type thymocytes [59, 60]. ThPOK is expressed in post-selection CD69^+^ DP thymocytes and in CD4SP cells and peripheral CD4^+^ T cells, but its expression is absent in CD8 lineage T cells [59, 60]. It has also been shown that MHC class II-induced signals lead to a high induction of ThPOK expression in CD69^+^ DP thymocytes, while MHC class I-signaled DP thymocytes remain ThPOK-negative, except for a small fraction of less than 5 % expressing only low levels of ThPOK [62–64]. Subsequent studies showed that ThPOK activity is also essential for maintaining CD4 lineage integrity in part by antagonizing *Cd4* silencer activity [64] and by repressing several CD8 lineage genes including Runx3 [63, 65]. In comparison, enforced expression of ThPOK in mature CD8^+^ T cells represses CD8 features leading also to the down-regulation of CD8 expression [66]. ThPOK has been shown to bind to several *Cd8* enhancers in CD4SP thymocytes [67]. Moreover, ThPOK interacts in vitro with class II histone deacetylases (HDACs) such as HDAC4/HDAC5 and the presence of ThPOK is required to recruit class II histone deacetylases to the *Cd8ab* gene complex [67]. This might lead to the repression of the *Cd8* loci during CD4 lineage differentiation. Together, these studies clearly demonstrate that ThPOK is a master CD4 lineage commitment factor that promotes CD4 lineage differentiation and represses CD8 lineage development of DP thymocytes and suggest that ThPOK also directly controls the *Cd4* and *Cd8* gene loci.

The importance of ThPOK for the development of CD4 lineage T cells also initiated studies to identify the *cis*-regulatory elements that direct *Thpok* expression in MHC class II-signaled thymocytes. Transgenic reporter expression assays and the analysis of how Runx complexes regulate ThPOK expression revealed the existence of several *cis*-regulatory elements essential for ThPOK expression and gene targeting approaches confirmed the importance of these *cis*-elements in the regulation of *Thpok* expression [64, 68, 69]. The proximal *Thpok* enhancer (designated PE), located about 3.6 kb downstream of exon 1A of the *Thpok* gene, directs expression in CD4SP and peripheral CD4^+^ T cells [68]. Interestingly, even though ThPOK expression was normally induced in PE-deficient thymocytes, ThPOK expression was gradually lost in PE-deficient CD4SP cells and CD4^+^ T cells, indicating that PE is essential for the up-modulation of ThPOK during CD4 lineage differentiation [64]. The other major *cis*-regulatory element is the *Thpok* silencer, located approximately 3 kb upstream of exon 1A of the *Thpok* gene. Deletion of the *Thpok* silencer results in derepression of ThPOK during CD8 lineage differentiation and as a consequence strong reduction in CD8^+^ T cell numbers [69]. Kappes and colleagues [68] identified another enhancer activity that is closely associated with the *Thpok* silencer region. In a recent study, Taniuchi and colleagues dissected this *cis*-regulatory element, designated as thymic enhancer (TE), from the *Thpok* silencer activity and showed that it acts as a positive regulatory element essential for the induction of ThPOK expression. Interestingly, Gata3 was found to be associated with TE, consistent with its role in inducing ThPOK expression discussed below [70]. Transgenic reporter assays also revealed the presence of one additional *cis*-element, termed general T-lymphoid element (GTE) that might be involved in the transcriptional maintenance of ThPOK expression [68], although its physiological role remains to be determined. For a detailed review about the transcriptional control of *Thpok* gene expression, see [57, 71].

### Runx complexes: regulators of *Cd4* silencing and *Cd8* gene expression

The Runx family (Runx1, Runx2, and Runx3) of transcription factors functions as heterodimers formed with the essential common cofactor CBFβ. Runx/CBFβ complexes play important roles in many developmental processes [72]. All three isoforms are known to be expressed in T cells [73] and the importance of Runx family proteins in thymocyte development and T cell lineage choice became clear when it was shown that Runx-binding sites within the *Cd4* silencer region are important for silencer function [74] and that Runx3 is important for the development and proper function of CD8 lineage T cells [73]. Runx proteins bind the *Cd4* silencer [74, 75] and are essential to silence CD4 expression in DN thymocytes and during CD8 lineage development. Runx1 association with the *Cd4* silencer is essential for repressing *Cd4* in DN cells, while Runx3 is needed to establish epigenetic silencing of the *Cd4* locus in CD8SP cells [73, 74]. Interestingly, CD8 lineage differentiation was impaired when mutating/deleting both Runx1 and Runx3 or CBFβ in T cells [69, 76]. At the molecular level, it was shown that Runx complexes bind to the *Thpok* silencer and are essential for repressing ThPOK in CD8 lineage T cells. In the absence of functional Runx complexes, ThPOK is derepressed, leading to the development of MHC class I-restricted CD4 lineage T cells [69].

Runx complexes also play an important role for CD8 expression, as they were found to be associated with *Cd8* enhancers in CD8^+^ T cells [75, 77]. However, Runx3-deficient mice display normal CD8 expression levels in naive CD8^+^ T cells [76, 78], therefore the molecular details of how Runx factors regulate CD8 expression during CD8 lineage development are not fully understood. As described in more detail below, a study from our laboratory demonstrated that Runx complexes are essential for maintaining *Cd8a* gene expression in activated CD8^+^ T cells [77], indicating a differential requirement of Runx3 in naive and activated/effector CD8^+^ T cells.

The observations that ThPOK represses Runx3 expression during CD4 lineage differentiation [63] while Runx complexes repress ThPOK during CD8 differentiation suggest that the interplay between Runx complexes and ThPOK in DP thymocytes determines CD4 vs. CD8 lineage choice and therefore Runx3 was considered as being the master regulator of the CD8 lineage. However, some observations indicate a slightly more complex mechanism. While transgenic overexpression of ThPOK is sufficient to silence Runx3 and to abrogate the development of CD8^+^ T cells, enforced Runx3 expression does not redirect MHC class II-restricted thymocytes into the CD8 lineage [79], suggesting that other factors potentially cooperate with Runx3 are essential for CD8 lineage development.

### MAZR: a regulator of CD8 expression in DN thymocytes and of CD4/CD8 cell-fate choice

One factor that might function in synergy with Runx complexes during CD8 lineage development is the BTB/POZ domain-containing transcription factor MAZR [56]. MAZR was initially identified as an interaction factor of the transcriptional repressor Bach2 [80]. We identified MAZR in a yeast one-hybrid screen as a transcriptional regulator that binds *Cd8* enhancer E8_II_ [55]. Subsequent ChIP assays revealed that MAZR is bound to several *Cd8* enhancers within the *Cd8ab* gene complex in DN thymocytes and that retroviral-mediated enforced expression of MAZR induces variegated expression of CD8 in DP thymocytes [55]. This indicates that MAZR acts as a repressor of CD8 expression during the DN to DP transition, probably via its interaction with N-CoR corepressor complexes [55], which are components of larger chromatin remodeling complexes such as Sin3A, NURD, and BAF [81–83]. MAZR expression is downregulated in DP cells and less MAZR is recruited to the *Cd8* loci compared to DN thymocytes [55]. In parallel, positive-acting factors and complexes might be induced during the DN to DP transition and recruited to the *Cd8* gene loci leading to a transcriptional “on” state facilitating CD8 expression. It is tempting to hypothesize that the change in the relative abundance of negative and positive-acting factors and chromatin-modifying complexes at the *Cd8* gene complex during the DN to DP transition determines the initiation of *Cd8a* and *Cd8b1* gene expression. In the absence of E8_I_/E8_II_, positive-acting factors are less efficiently recruited to the *Cd8ab* gene complex. Therefore negative factors are more abundant and CD8 expression is impaired in DP thymocytes. If MAZR expression is enforced, negative-acting factors are more abundantly recruited and hence CD8 expression is variegated. This model is supported by the observation that the deletion of MAZR in E8_I_/E8_II_-deficient thymocytes restores CD8 expression [84], similar to what is observed upon deletion of DNA methyltransferase 1 (Dnmt1) on an E8_I_/E8_II_-deficient background, since the relative balance between negative- and positive-acting factors might be again shifted towards a transcriptional “on” state.

Moreover, the analysis of MAZR-deficient mice revealed that MAZR is part of the transcription factor network controlling CD4/CD8 cell-fate choice. MAZR is bound to the *Thpok* silencer and essential to repress ThPOK in MHC class I-signaled thymocytes [84]. In the absence of MAZR, a fraction of MHC class I-signaled DP cells derepresses ThPOK, resulting in redirection into CD4 lineage T cells. In addition, MAZR was found to interact with Runx complexes, which might indicate synergistic repression of ThPOK [84] and potentially also synergistic activities in mediating CD8 lineage differentiation. Finally, MAZR-deficient mice are smaller in size and are born at reduced Mendelian frequencies due to impaired embryonic development [84, 85]. Moreover, MAZR has been implicated to function as a tumor suppressor gene [86] and MAZR-null male mice are infertile [84, 87]. Thus, MAZR also has important roles in other cell lineages beyond the hematopoietic system.

### Ikaros, Mi-2β, and AP4: additional factors regulating *Cd4* silencer activity

Mi-2β, an ATP-dependent chromatin remodeling factor [88] and Ikaros, a transcriptional regulator with many functions during lymphocyte development that also associates with Mi-2β [89] and other chromatin remodeling complexes [90, 91], were shown to regulate CD4 expression during early stages of thymocyte development. Loss of Ikaros leads to *Cd4* derepression in DN thymocytes [89], while Mi-2β deficiency leads to the emergence of DP cells that display strongly reduced CD4 expression levels [89]. Interestingly, mice lacking both factors displayed a reversal of both phenotypes, thus revealing an antagonistic interplay between Mi-2β and Ikaros in controlling *Cd4* gene expression during the DN to DP transition. While the association of Ikaros with the *Cd4* silencer mediates *Cd4* repression in DN cells, the simultaneous binding of Ikaros and Mi-2β in DP thymocytes leads to the inactivation of *Cd4* silencer activity, in part by Mi-2β-mediated recruitment of histone acetyltransferases [89].

One additional transcription factor involved in the regulation of *Cd4* silencing is the basic helix-loop-helix (bHLH) ZIP protein AP4, which was found to be necessary for efficient *Cd4* repression both in DN thymocytes as well as in memory CD8^+^ T cells [92]. AP4 binds to E4_P_ but not to the *Cd4* promoter or *Cd4* silencer and interacts with Runx1 in cells that have silenced CD4 expression. AP4-deficient mice show *Cd4* derepression in DN cells. Moreover, CD4 expression is modulated in AP4-deficient memory CD8^+^ T cells that contain a mutated *Cd4* silencer allele, indicated by an increase in the numbers of CD8 effector T cells that express CD4 in comparison to cells carrying the mutant *Cd4* silencer only [92]. Thus, AP4 is another factor that contributes to *Cd4* silencing both in DN thymocytes and in CD8^+^ T cells.

### The bHLH factors HEB and E2A control the activity of the *Cd4* enhancer E4_P_

Soon after the discovery of the *Cd4* enhancer E4_P_, experiments were initiated to identify transcription factors that interact with this *cis*-regulatory element. The bHLH proteins HEB and E2A were found to bind to E4_P_ [93] and gene-targeting experiments revealed that HEB is essential for the up-regulation of CD4 expression in DP thymocytes, since loss of HEB leads to the generation of increased CD4^lo^CD8^+^ and reduced CD4^+^CD8^+^ DP thymocyte subsets [94]. Interestingly, HEB-deficient mice display normal CD4 expression in peripheral T cells, although T helper cell numbers were reduced in comparison to wild-type mice [94]. This is in part reminiscent of the phenotype observed in E4_P_-deficient mice [35]. In addition, mice with a combined heterozygosity for HEB and E2A displayed a similarly reduced expression of CD4, indicating that CD4 expression is sensitive to the expression levels of the bHLH factors HEB and E2A [94, 95]. The combined deletion of HEB and E2A in T cells (using *Cd4*-*Cre*) revealed that both proteins are required to maintain thymocytes at the DP stage until a functional T cell receptor is expressed, since HEB/E2A-null DP cells can progress to the SP stage even without a TCR signal [96]. However, the majority of SP cells in the absence of HEB/E2A were CD8^+^ cells, indicating that HEB and E2A are essential for CD4 lineage development [96]. Consistently, deletion of the E-protein inhibitors Id2 and Id3 allowed CD4 lineage development but blocked the generation of CD8 lineage T cells [97]. Furthermore, the analysis of ThPOK and Gata3 expression in HEB/E2A-null DP thymocytes suggest that HEB/E2A function upstream of CD4 lineage specification [97].

### SATB1, Bcl11b, and Ikaros: transcription factors implicated in the regulation of *Cd8* gene expression

SATB1 (special AT-rich binding protein) is a matrix attachment region (MAR) binding protein that can recruit chromatin remodeling complexes to SATB1 target sites [98, 99]. At the *Cd8ab* gene complex, SATB1 binds to a MAR designated L2a within *Cd8* enhancer E8_V_ in DP and CD8SP thymocytes [100]. SATB1-deficient mice display a severe block at the DP stage of T cell development [101]. Mice with a knockdown mutation of SATB1 (via T cell-specific expression of antisense *Satb1*) display reduced percentages of CD8SP thymocytes and T cells, although CD8 expression levels were not altered in SATB1 antisense thymocytes [102]. However, a closer in vitro analysis of SATB1-deficient as well as SATB1-antisense DP thymocytes revealed that SATB1 binds to E8_III_ and that SATB1 is essential to re-express CD8 in signaled (PMA/ionomycin) DP thymocytes in response to IL-7 [103]. This suggests an important positive role for SATB1 in the regulation of CD8 expression during CD8 lineage development, although the molecular details of how SATB1 mediates the activation of the *Cd8* loci are not yet known.

The zinc-finger transcription factor Bcl11b plays multiple roles during T cell development. Germline deletion of Bcl11b initially demonstrated a crucial role for this factor in *Tcrb* recombination and β-selection, resulting in a block at the DN stage in the absence of Bcl11b [104]. Conditional deletion of Bcl11b using either *Cd4*-*Cre* or *Lck*-*Cre* revealed additional roles for Bcl11b for the survival of DP thymocytes as well as positive selection, since conditional Bcl11b knockout mice showed a developmental block at the DP stage [105, 106]. Bcl11b-deficient DP (CD4^+^CD8^+^CD3^lo^) cells displayed altered expression of more than 1,000 genes, and *Thpok* as well as *Runx3* were found to be up-regulated in the absence of Bcl11b. Moreover, ChIP-seq approaches revealed that Bcl11b binds to several regions within the *Thpok* and *Runx3* gene loci, suggesting direct repression of ThPOK and Runx3 by Bcl11b in DP thymocytes [106]. The role of Bcl11b in controlling CD8 expression only became clear when it was conditionally ablated in mature CD8^+^ T cells (using distal *Lck*-*Cre*). Bcl11b is associated with the *Cd8* enhancers E8_I_, E8_IV_, and E8_V_ and Bcl11b is necessary to maintain CD8 expression in mature CD8^+^ T cells during bacterial infection as well as during in vitro activation [107].

Beside its role in the regulation of *Cd4* gene expression (as discussed above), Ikaros has also been implicated in the control of the *Cd8ab* gene complex. Kioussis and colleagues [108] showed that Ikaros is associated in vivo with *Cd8* enhancers E8_I_ and E8_V_. Ikaros appears to have a positive regulatory activity for the *Cd8* loci, since reduced levels of Ikaros (in *Ikzf* ^+*/*−^ mice) lead to an increase in the variegated expression of a reporter gene driven by *Cd8* enhancers. In addition, mice heterozygous for Ikaros and deficient for the Ikaros family member Aiolos showed impaired activation of the *Cd8ab* gene complex resulting in the development of “CD8-negative” DP thymocytes [108], similar to the phenotype observed in E8_I_/E8_II_- [49] E8_II_/E8_III_- [51], and E8_V_-deficient [50] thymocytes or in BAF complex mutant mice [54].

### Other transcription factors involved in CD4/CD8 cell-fate choice

Additional transcription factors implicated in the regulation of CD4/CD8 lineage differentiation are Tox, Gata3 and c-Myb. Overexpression of Tox, a member of the high-mobility group box protein family, leads to an enlarged CD8SP thymocyte pool at the expense of CD4SP cells, which might be due to enhanced Runx3 expression in Tox transgenic mice [109]. In Tox-deficient mice, thymocyte development is partially blocked at the CD4^lo^CD8^lo^ stage and CD4SP generation is completely abolished, whereas some CD8SP cells still develop [110]. Interestingly, ThPOK is not expressed in CD4^lo^CD8^lo^ thymocytes of Tox-deficient TCR transgenic mice, indicating that Tox might carry out its role in CD4 lineage differentiation upstream of ThPOK [110]. The zinc finger protein Gata3 is another transcription factor shown to be important for the generation of CD4^+^ T cells during thymocyte differentiation. In conditional Gata3 knockout mice, the numbers of CD4SP cells were strongly reduced, whereas CD8SP cells were unperturbed [111]. A subsequent study found that this might be partially explained by the observation that Gata3 binds the *Thpok* locus at a regulatory site necessary for its expression and that the induction of ThPOK expression depends on Gata3 [62]. However, enforcing ThPOK expression cannot compensate for loss of Gata3 [62]. Moreover, Gata3 represses Runx3 expression and binds the *Runx3 *locus in vivo [112]. This indicates that Gata3 is not only important for the induction of CD4 lineage genes but also for the repression of CD8 lineage genes. Another transcription factor implicated in CD4/CD8 lineage choice is c-Myb, which has been shown to have multiple functions during thymocyte development. c-Myb is essential for developmental progression through the DN3 stages, for the survival of preselection DP cells and for the differentiation of CD4SP thymocytes [113]. The phenotype of conditional c-Myb-deficient thymocytes resembles in part the phenotype of Gata3-deficient thymocytes, i.e., impaired CD4 lineage development and unperturbed CD8 lineage development [113, 114]. This was attributed to the ability of c-Myb to bind and to regulate the *Gata3* locus, since c-Myb-deficient thymocytes display reduced expression of Gata3 [114]. This suggests that c-Myb acts upstream of Gata3 and potentially also upstream of other CD4 lineage specification and commitment factors. However, there is no indication that Tox, GATA3, or c-Myb are directly involved in the regulation of *Cd4*, *Cd8a*, and *Cd8b1* gene expression.

## Differential regulation of coreceptor gene expression between naive and effector/memory T cells

The discovery of the *cis*-regulatory networks directing *Cd4* and *Cd8* coreceptor gene expression provided important insights into transcriptional control mechanisms in T cells. The identification of enhancers that direct expression only either in immature DP cells or in mature T cells indicated that different *cis*-elements are required at distinct developmental stages. However, subsequent studies demonstrated a differential requirement for enhancers also within mature T cell subsets. The first experimental evidence came from studies that showed that E4_P_ is not sufficient to maintain transgene expression after activation of mature CD4^+^ T cells [115], whereas the expression of the endogenous *Cd4* gene is maintained upon activation. This suggests that the expression of CD4 is differentially regulated between naive and effector/memory CD4^+^ T cells. Moreover, this finding implies the existence of an additional *Cd4* enhancer specifically required for effector/memory CD4^+^ T cells, however the molecular identity of this *cis*-element remains to be determined.

Our laboratory revealed that a transcriptional program that is distinct from naive T cells regulates *Cd8a* gene expression during CD8^+^ effector T cell differentiation and that *Cd8* enhancer E8_I_ and Runx/CBFβ complexes are required for CD8 expression in activated CD8^+^ T cells [77]. This finding was initiated by the observation that subsets of CD8αβ^+^ T cells transiently express CD8αα homodimers in an E8_I_-dependent manner upon activation [116]. Cheroutre and colleagues [116] linked CD8α expression on CD8αβ^+^ T cells to the survival and differentiation of memory precursor cells into memory cells and reported impaired memory formation in E8_I_-deficient mice. However, it was also shown that memory responses can occur in the absence of CD8α expression in E8_I_-deficient mice [117, 118]. Interestingly, in one of the studies Kaech and colleagues [117] observed a decrease in CD8αβ expression levels on splenic T cells in LCMV-infected E8_I_-deficient mice. This provided the first indication that E8_I_-deficient activated CD8^+^ T cells have a defect in CD8 expression upon activation. Subsequent studies revealed mechanistic insight into how CD8 expression is regulated in naive versus activated effector T cells. E8_I_-deficient mice down-regulated CD8α upon activation and this correlated with enhanced repressive histone marks at the *Cd8a* promoter in the absence of E8_I_, while *Cd8b1* expression levels and histone marks at the *Cd8b1* promoter region remained unaffected [77]. The treatment of E8_I_-deficient CD8^+^ T cells with the HDAC inhibitor trichostatin A blocked the down-regulation of CD8α expression, suggesting epigenetic control of the *Cd8a* gene locus in activated CD8^+^ T cells [77]. Moreover, Runx/CBFβ complexes bound the *Cd8ab* gene cluster in activated CD8^+^ T cells, and *Cd8a* gene expression was down-regulated in Runx3- or CBFβ-deficient CD8^+^ T cells as observed in E8_I_-deficient T cells. This strongly suggests direct control of the *Cd8a* locus by Runx complexes during CD8^+^ T cell activation. Remarkably, CD8^+^ effector T cells maintained high levels of CD8α when CBFβ was conditionally deleted after CD8^+^ T cells were activated [77]. Thus, naive and effector CD8^+^ T cells utilize different *cis*-regulatory elements and mechanisms to regulate *Cd8a* gene expression. While multiple *cis*-elements that in part might have redundant functions drive CD8 expression in naive T cells, there is E8_I_- and Runx3/CBFβ-dependent epigenetic programming of the *Cd8a* locus during CD8^+^ T cell activation. The observation that *Cd8a* expression can be maintained in the absence of E8_I_/Runx complexes is reminiscent to the above described study demonstrating that CD4 expression remained at high levels when E4_P_ was deleted in mature T helper cells [35]. Together, these data indicate epigenetic regulation of coreceptor gene expression in mature T cells.

Interestingly, a recent study suggests a crucial role for the *Cd8* enhancer E8_I_ in directing *Cd8a* gene expression in a distinct CD4^+^CD8αα^+^ effector T cell subset with CTL features that is localized in the gut [119]. It has been shown that some conventional CD4^+^ T cells, despite their initial commitment to the CD4^+^ helper fate and the expression of MHC class II-restricted αβTCRs, can get functionally reprogrammed and acquire CTL features [119, 120]. This reprogramming is induced in some CD4^+^ T cells due to activation-induced loss of ThPOK expression, which also leads to a *Cd8* enhancer E8_I_-dependent induction of *Cd8a* but not of *Cd8b1* gene expression and thus the generation of CD4^+^CD8αα^+^ effector T cells [119]. Interestingly, it has also been shown that TGFβ can induce CD8α expression on conventional CD4^+^ T cells [121]. Notably, E8_I_-deficient mice displayed reduced CD8αα on IELs [44, 46] and a much lower percentage of CD4^+^CD8αα^+^ IELs [119]. Together, these findings support the idea that E8_I_ is a crucial enhancer required in peripheral T cells for the modulation of *Cd8a* gene expression.

## Regulation of the spatial organization of the *Cd4* and *Cd8* loci

In addition to the above-described interactions between *cis*-regulatory elements and transcription factors, the spatial organization of chromosomes and gene loci and the communication between *cis*-regulatory elements is also important for the proper transcriptional regulation of coreceptor gene expression during CD4/CD8 lineage differentiation. Merkenschlager and colleagues [122] as well as Robey and colleagues [123] showed that in MHC class II-selected DP thymocytes that become CD4SP cells the *Cd8* gene loci are relocated to centromeric heterochromatin areas where gene transcription is stably silenced. In contrast, in DP cells that are positively selected on MHC class I to develop into CD8 lineage cells, the *Cd4* gene was found to be associated with heterochromatin at this stage. Thus, the repositioning of *Cd4* and *Cd8* coreceptor alleles within the nucleus is predictive for CD4/CD8 lineage choice [122, 123].

The nuclear position of the *Cd8* loci relative to its chromosomal territory (CT) during T cell development has been analyzed in more detail by Kioussis and colleagues [124]. Using three-dimensional fluorescent in situ hybridization (3D FISH) technology, the authors showed that the *Cd8* genes are in close proximity to the center of their CT in CD8 non-expressing cells (DN, CD4SP), while the *Cd8* genes are relocated outside their CT centers in cells that actively express CD8 (DP, CD8SP). Moreover, by performing chromosome conformation capture (3C) assays, a frequent physical contact between *Cd8* enhancers and the *Cd8a* promoter was detected in CD8 expressing cells. In contrast, in CD8 non-expressing cells this interaction was significantly decreased [124]. These observations suggest that the movement of the *Cd8* loci outside its CT leads to a spatial clustering of *cis*-regulatory elements and the formation of a chromatin hub that facilitates *Cd8* gene expression.

An example of gene regulation via chromatin looping that facilitates the interaction between *cis*-regulatory elements is the regulation of the *Cd4* gene locus during the progression from the DN to the DP stage. Runx1 promotes the interaction between the *Cd4* silencer and the proximal *Cd4* enhancer E4_P_ in DN cells [125]. Upon differentiation to the DP stage Runx1 expression decreases allowing the elongation factor P-TEFb to interact with RNAPII, which leads to the formation of a productive chromatin loop that enables *Cd4* gene expression.

A study published by Skok and colleagues [126] provided new mechanistic insight into the long-range control of *Cd4* and *Cd8* expression by distant enhancers, silencer, and promoters. Using 3D FISH combined with confocal laser scanning microscopy, the authors showed that the interchromosomal association of the *Cd4* and *Cd8* gene loci is a crucial mechanism that controls the regulated expression of coreceptors during T cell development. When measuring the distance between *Cd4/Cd8* loci in murine thymocytes at different developmental stages (DN, DP, CD4^+^CD8^lo^, CD4SP and CD8SP), the strongest physical interaction between the *Cd4/Cd8* loci was observed in cells that expressed CD8 (DP and CD8SP). Thus, the close association of the *Cd4* and *Cd8* loci correlates with activated *Cd8* gene transcription in DP and CD8SP cells. This suggested that genomic elements regulating *Cd8* gene transcription might be involved in influencing the *Cd4/Cd8* loci association. To test this hypothesis, the association of *Cd4/Cd8* loci in *Cd8* enhancer E8_I_/E8_II_-deficient DP thymocytes was assessed. As described above, a fraction of E8_I_/E8_II_ DP thymocytes did not express CD8 (and hence were referred as “CD8-negative” DP cells) due to impaired up-regulation of CD8 during the DN to DP transition [55]. In “CD8-negative” E8_I_/E8_II_ DP thymocytes, there was a significantly reduced *Cd4*-*Cd8* interaction accompanied by a strong reduction in *Cd8* gene transcription in comparison to CD8 expressing E8_I_/E8_II_-deficient DP thymocytes [126]. Consistently, *Cd8* loci association with pericentromeric heterochromatin was observed at an increased percentage in “CD8-negative” E8_I_/E8_II_-null DP cells correlating with an epigenetic “off” state of the *Cd8* locus. In summary, these findings suggest a crucial role for E8_I_/E8_II_ in regulating *Cd4/Cd8* loci association. These studies also suggest that during thymocyte development CD4/CD8 cell-fate choice might be in part implemented by the physical interaction between the *Cd4* and *Cd8* genes mediated by *cis*-regulatory sequences. However, the exact mechanism of how *cis*-regulatory elements and gene loci find each other in chromatin hubs remains elusive. It is conceivable that *trans*-acting proteins are involved in this process, since they provide binding specificity and also mediate protein–protein interactions. ThPOK and Runx proteins are promising candidate factors for the regulation of *Cd4*/*Cd8* loci association, since they control CD4/CD8 cell-fate choice and the mutual exclusive expression of CD4 and CD8 coreceptor molecules in mature T cells. Indeed, Runx3 mediates the association of the *Cd4* and *Cd8* loci by binding to the *Cd4* silencer and predominantly to E8_I_, whereas the binding of ThPOK to the *Cd4* silencer reverses the association of both loci [126]. While the close association between the *Cd4* and *Cd8* gene loci favors the expression of the CD8 coreceptor molecule in DP and CD8SP, the disruption of this interaction by ThPOK leads to the termination of *Cd8* expression during CD4 lineage development. This raises the exciting hypothesis that ThPOK and Runx3, essential factors for CD4/CD8 cell-fate choice, also control the association of the *Cd4* and *Cd8* gene loci. Collectively, these studies suggest that the generation of chromatin loops mediated by antagonizing transcription factors might control the expression of coregulated genes.

## Conclusions

Research performed during the last two decades revealed comprehensive mechanistic insight into the developmental stage- and subset/lineage-specific regulation of CD4 and CD8 coreceptor expression and also into the interchromosomal crosstalk between the *Cd4* and *Cd8* gene loci. These studies thus provided important insight into transcriptional control mechanisms during T cell development and in peripheral effector T cells in general. Moreover, the identification of transcription factors involved in the transcriptional regulation of CD4 and CD8 significantly advanced the knowledge about the transcription factor network regulating CD4/CD8 cell-fate choice of DP thymocytes.

Although the *Cd4* and *Cd8* gene loci represent probably one of the best-characterized gene loci in T cells, several important questions remain to be addressed. Which *cis*-regulatory element at the *Cd4* locus represents the “maturation” enhancer and how is CD4 expression maintained in CD4^+^ effector T cells? How is *Cd8* gene expression (epigenetically?) maintained in naive CD8^+^ T cells in the combined absence of E8_I_ and E8_II_? Moreover, the *cis*-elements required to direct expression of CD4 or CD8α in DCs have not been identified. It is also not known whether a unique negative-acting *cis*-regulatory element (*Cd8* “silencer”) or repressive transcription factors (targeting potentially several *cis*-elements) are required to shut off CD8 expression during CD4 T cell lineage differentiation. Finally, future studies using ChIP-seq approaches are also essential in understanding how the recruitment of transcription factors to the *Cd4* and *Cd8* gene loci at different developmental stages and in effector T cells is regulated. This could reveal whether there is a hierarchy in the recruitment of transcription factors and chromatin remodeling complexes and how the recruitment of these factors is linked to epigenetic regulation of coreceptor expression. It can be expected that answers to these questions will provide novel insight into transcriptional control mechanisms in T cells and will also lead to a better understanding of how CD4/CD8 lineage differentiation is regulated during T cell development.
